# Genome-Wide Transcriptome Directed Pathway Analysis of Maternal Pre-Eclampsia Susceptibility Genes

**DOI:** 10.1371/journal.pone.0128230

**Published:** 2015-05-26

**Authors:** Hannah E. J. Yong, Phillip E. Melton, Matthew P. Johnson, Katy A. Freed, Bill Kalionis, Padma Murthi, Shaun P. Brennecke, Rosemary J. Keogh, Eric K. Moses

**Affiliations:** 1 Department of Perinatal Medicine, Pregnancy Research Centre and The University of Melbourne, Department of Obstetrics and Gynaecology, The Royal Women’s Hospital, Locked Bag 300, Corner Grattan Street and Flemington Road, Parkville, 3052, Victoria, Australia; 2 Centre for Genetic Origins of Health and Disease, The University of Western Australia, 35 Stirling Highway, Crawley, 6009, Western Australia, Australia; 3 Department of Genetics, Texas Biomedical Research Institute, P.O. Box 760549, San Antonio, Texas, United States of America; University of Arkansas for Medical Sciences, UNITED STATES

## Abstract

**Background:**

Preeclampsia (PE) is a serious hypertensive pregnancy disorder with a significant genetic component. Numerous genetic studies, including our own, have yielded many susceptibility genes from distinct functional groups. Additionally, transcriptome profiling of tissues at the maternal-fetal interface has likewise yielded many differentially expressed genes. Often there is little overlap between these two approaches, although genes identified in both approaches are significantly associated with PE. We have thus taken a novel integrative bioinformatics approach of analysing pathways common to the susceptibility genes and the PE transcriptome.

**Methods:**

Using Illumina Human Ht12v4 and Wg6v3 BeadChips, transcriptome profiling was conducted on n = 65 normotensive and n = 60 PE *decidua basalis* tissues collected at delivery. The R software package libraries *lumi* and *limma* were used to preprocess transcript data for pathway analysis. Pathways were analysed and constructed using Pathway Studio. We examined ten candidate genes, which are from these functional groups: activin/inhibin signalling—*ACVR1*, *ACVR1C*, *ACVR2A*, *INHA*, *INHBB;* structural components—*COL4A1*, *COL4A2* and M1 family aminopeptidases—*ERAP1*, *ERAP2 and LNPEP*.

**Results/Conclusion:**

Major common regulators/targets of these susceptibility genes identified were AGT, IFNG, IL6, INHBA, SERPINE1, TGFB1 and VEGFA. The top two categories of pathways associated with the susceptibility genes, which were significantly altered in the PE decidual transcriptome, were apoptosis and cell signaling (p < 0.001). Thus, susceptibility genes from distinct functional groups share similar downstream pathways through common regulators/targets, some of which are altered in PE. This study contributes to a better understanding of how susceptibility genes may interact in the development of PE. With this knowledge, more targeted functional analyses of PE susceptibility genes in these key pathways can be performed to examine their contributions to the pathogenesis and severity of PE.

## Introduction

Preeclampsia (PE) is a common and serious pregnancy complication characterised by new-onset hypertension and proteinuria after 20 weeks’ gestation and affects between 2–8% of all pregnancies worldwide [1]. Although the disorder has been known since antiquity, the cause of PE remains elusive with the only known cure being the removal of the placenta [2]. Due to the severity of the mother’s condition, there is often an urgent need to deliver the fetus prematurely. PE accounts for over 40% of medically indicated pre-term births [3]. Preterm births are associated with greater neonatal morbidity and mortality in the short term, as well as high economic, health and social costs later in life [4, 5]. A PE mother is also at increased long term risk of developing ischemic heart disease, stroke and cardiovascular disease [6–8]. Therefore, the consequences of PE are not merely short term but may have persistent, long term effects on the mother and child.

PE susceptibility is influenced in part by a strong genetic component. Heritability estimates range from 0.31 to 0.54 [9–11], and numerous candidate genes have been implicated either by linkage or association methods. The *ACVR2A* [12] and *STOX1* [13] genes were identified by genetic linkage methods. Several genes (*AGT*, *ACE*, *AGTR1*, *AGTR2*, *FV*, *MTHFR*, *NOS3* and *TNFα*) have been the focus of candidate gene association studies [14]. Genome-wide association scans have implicated multiple genetic loci [15], including the *INHBB* [16], and *PSG11* loci [17]. It is widely accepted that PE does not follow a Mendelian inheritance except in a few families [18]. Instead, PE is the result of complex interactions between the maternal and fetal genotypes and environment factors.

Another approach used to investigate the pathogenesis of PE is a microarray study design to identify differentially expressed genes in tissues at the maternal-fetal interface and thereby gain an insight into possible disease mechanisms. Placental [19, 20] and decidual [21–25] tissue microarray studies have reported numerous differentially expressed genes. However, there is often little concordance between microarray studies, likely due to factors such as insufficient power and heterogeneity of tissue samples [20, 26]. To date, most transcriptome studies have been conducted in the fetal-derived placenta with only a few using maternal-derived decidua [19–25, 27]. Conversely, most genetic linkage/association studies have focused on the maternal genotype [11–13, 16, 28–31]. Taken together, this discordance in study design strategies partly explains the very small overlap between genetic association/linkage and expression studies. Hence, further expression studies should focus on the maternal tissue in addition to the fetal tissue.

Whilst the aforementioned study designs have reported numerous PE candidate genes, there is frustratingly little overlap in the genes identified. A recent study by Founds [32], linking placental global gene expression with PE susceptibility loci, showed that 40% of genes altered in first trimester placental chorionic villus sampling resided in known PE susceptibility loci. However, these account for only 13 genes, a small fraction of known PE candidate genes. A review by Jebbink *et al*. [33] showed at least 178 genes associated with PE have been identified by both the candidate gene and genome-wide study approaches, with many more genes identified since then. It is unlikely that there will be a single, universal causative gene for PE. Given the large diversity of genes identified from genetic studies and microarray studies, we have taken a novel integrative bioinformatics approach to bridge this gap by analysing pathways common between susceptibility genes identified by the genetic association approach and the PE transcriptome. Identifying common underlying biological pathways will allow us to perform more specific and targeted functional analyses to address the complexities of PE.

Our earlier studies showed that maternal susceptibility genes, which are from various functional groups: activin/inhibin signalling—*ACVR1*, *ACVR1C*, *ACVR2A*, *INHA*, *INHBB*; structural components—*COL4A1*, *COL4A2* and M1 family aminopeptidases—*ERAP1*, *ERAP2* and *LNPEP*, were differentially expressed in tissues at the maternal-fetal interface [12, 34]. Functional studies of these genes are presently limited and based on the assumptions of how PE is thought to develop. Hence, to identify unbiased, novel functional roles of these susceptibility genes, we performed a genome-wide transcriptome directed pathway analysis of maternal PE susceptibility genes. By taking into account the underlying pathways of the whole genome instead of focussing specifically on differentially expressed genes, we aimed to use an integrative bioinformatics approach identify novel biological processes involved in the development of PE.

## Materials and Methods

### Patient Samples

*Decidual basalis* samples were collected from a total of n = 65 normotensive and n = 60 PE patients at Caesarean section as described previously [12]. Normotensive patients underwent Caesarean section due to breech presentation, maternal request or previous history. PE was defined according to the Australasian Society for the Study of Hypertension in Pregnancy criteria [35, 36]. Exclusion criteria for PE patients included diabetes and systemic lupus erythematosus. Blood pressures of normotensive patients were recorded as <140/90 mmHg. A non-treating obstetrician independently verified patient clinical records. Tissue samples were verified as decidual by hospital pathologists. Each patient gave written informed consent for samples to be used for the study. Research and ethics approval was granted by The Royal Women’s Hospital Research and Ethics Committees, Melbourne, Australia and the Institutional Review Board of the University of Texas Health Science Center at San Antonio, Texas, USA.

### Sample Processing

Harvested decidual tissue was placed into an appropriate volume of RNA-later (Qiagen, Hilden, Germany) and kept at 4°C for at least 24 hrs. Up to 250 mg of decidual tissue was then removed from the RNA-later and stored at -80°C. Total RNA was extracted from decidual samples using RNeasy Midi kits (Qiagen) with yield and quality determined as described previously [12]. Complementary RNA synthesis, amplification and purification were performed as described previously [24].

### Transcriptional Profiling

Microarray interrogation of decidual complementary RNA was performed in two batches. The first batch of n = 23 normotensive and n = 25 PE samples were hybridised onto Illumina HumanWG-6 v3 Expression BeadChips (Illumina Inc., San Diego, CA, USA), while the second batch of n = 42 normotensive and n = 35 PE samples were hybridised onto Illumina HumanHT-12 v4 Expression BeadChips (Illumina Inc.) in accordance with Illumina’s Whole-Genome Gene Expression Direct Hybridisation assay protocol. All samples were scanned on the Illumina iScan System with iScan Control Software (v3.2.45). Illumina’s GenomeStudio software (v2010.2), Gene Expression Module (v1.7.0), was used to generate a control summary report to assess assay performance and quality control metrics. Updates in array content, from one BeadChip version to another, often results in changes in transcript probe identifiers. We therefore utilised PROBE_SEQUENCE information as the unique identifier to highlight transcript probes common to both the HumanWG-6 and HumanHT-12 BeadChips. A total of 39,426 common probes were identified for data pre-processing. To account for batch effects, the data from each batch was analysed independently. The raw microarray data are accessible at the Gene Expression Omnibus repository with the Accession Number GSE60438 (National Center for Biotechnology Information, National Institutes of Health, Bethesda, MD, USA).

### Data Pre-processing

Background noise was subtracted from transcript data for analysis using Illumina’s GenomeStudio software (v2010.2), Gene Expression Module (v1.7.0). The data from each batch were then pre-processed independently with the open source software R version 3.0.2 available via www.bioconductor.org. The lumi R package [37] was used to log2-transform and quantile normalise the data. The limma R package [38] was then used to rank differential gene expression with moderated *t* tests.

### Pathway Analyses

The list of ranked genes for each microarray batch and the list of susceptibility genes were imported into Pathway Studio 9.0 (Elsevier, Amsterdam, Netherlands) for pathway analysis. Gene set enrichment analysis (GSEA) was performed on gene expression data to identify altered pathways throughout the genome. To determine the pathways associated with the susceptibility genes, a sub-enrichment analysis was performed on the list of susceptibility genes. Pathways are represented by the Gene Ontology (GO) set class of biological processes. Data files were then exported as databases in Microsoft Access 2010 (Microsoft Corp., Redmond, WA, USA) to first determine the consistently altered pathways between the two transcriptome profiling batches and then the concordant pathways between susceptibility genes and the PE transcriptome.

### Gene Network Construction

To determine the interactions between susceptibility genes from the different functional groups, gene networks were constructed. The literature-based ResNet Mammalian 9.0 Database in Pathway Studio 9.0 (Elsevier) was used to determine common pathway targets and regulators of the susceptibility genes. Pathways were selected using their expression relationship. Each pathway link is supported by at least one published reference. The references for each link were manually cross-checked to remove any erroneous links.

### Statistical Analyses

Student’s *t* test with Welch’s Correction and 2 × 2 contingency table with Fisher’s Exact Test were used for analysing patient characteristics where appropriate on GraphPad Prism 5 (GraphPad Software Inc., La Jolla, CA, USA). A value of p < 0.05 was considered statistically significant for patient characteristics. Mann-Whitney *U* test was used for enriching significant pathways in the GSEA on Pathway Studio 9.0. For the pathway analyses, a semi-conservative value of p < 0.001 was selected as the statistical cut-off to maximise the identification of novel pathways, while minimising the number of potential false positives in multiple testing.

## Results

### Patient Characteristics

Gestational age, infant birth weight, birth weight percentiles, gravidity and parity between the n = 65 normotensive and n = 60 PE patients were significantly different (Table 1). No significant difference was observed for maternal age or infant sex. The significant differences for gravidity and parity were expected given that PE is more common in first pregnancies. The lower birth weights and gestational age at delivery for the PE patients are consistent with earlier delivery due to the severity of the disease.

**Table 1:** Summary of patient characteristics.

Patient characteristics^a^	Normotensive (n = 65)	Pre-eclamptic (n = 60)	p-value^b^
Maternal age (years)	32.3±0.5	31.2±0.8	0.24
Gestational age (weeks)	39.1±0.1	32.3±0.5	<0.001
Infant sex^c^	33F, 32M	27F, 34M	0.48
Infant birth weight (g)	3369.2±54.4	1738. 1±125.3	<0.001
Infant weight percentiles (%)^d^	25–50	10–25	<0.001
Gravidity	29 primi-, 36 multi-	40 primi-, 20 multi-	<0.001
Parity	32 primi-, 33 multi-	51 primi-, 9 multi-	<0.001
Systolic blood pressure (mmHg)	<140	172.2±1.9	NA
Diastolic blood pressure (mmHg)	<90	105.6±1.1	NA
Antihypertensive treatment(s)	Not given	48	NA
MgSO_4_ treatment	Not given	37	NA

NA, not applicable.

^a^ Shown is the mean ± SEM unless stated otherwise.

^b^ Student’s *t* test with Welch’s Correction for parametric data and 2 × 2 contingency table with Fisher’s Exact Test for categorical data were used.

^c^ One PE patient had a twin pregnancy.

^d^ Data shown as median.

### Common pathways of susceptibility genes

**Fig 1:**
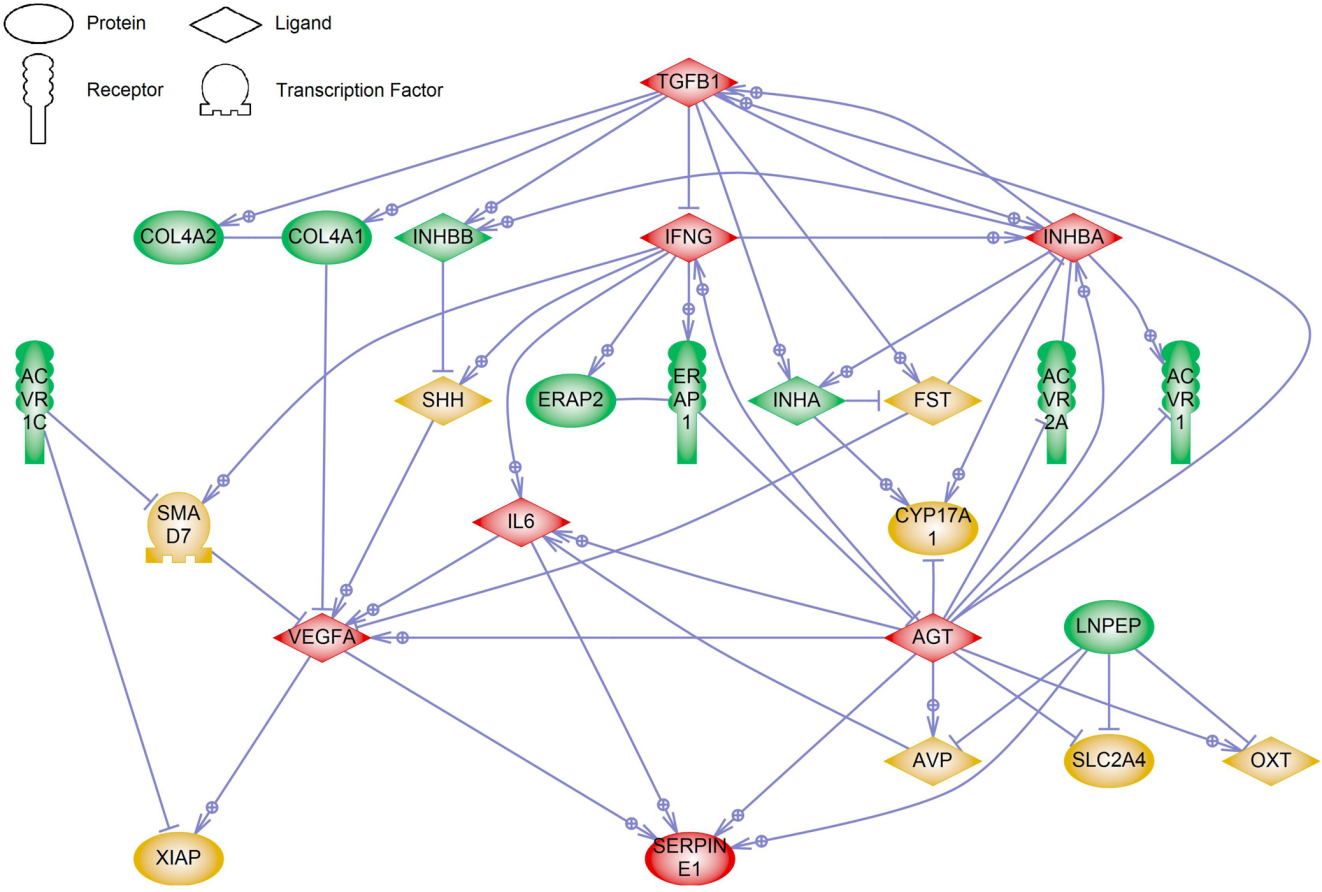
Common regulators and targets of maternal PE susceptibility genes. A gene network showing the interactions between the maternal PE susceptibility genes was generated with Pathway Studio 9.0. Each link is supported by at least one published reference. Maternal PE susceptibility genes investigated are coloured in green, connecting genes in yellow and major regulator/target genes in red.

**Fig 2:**
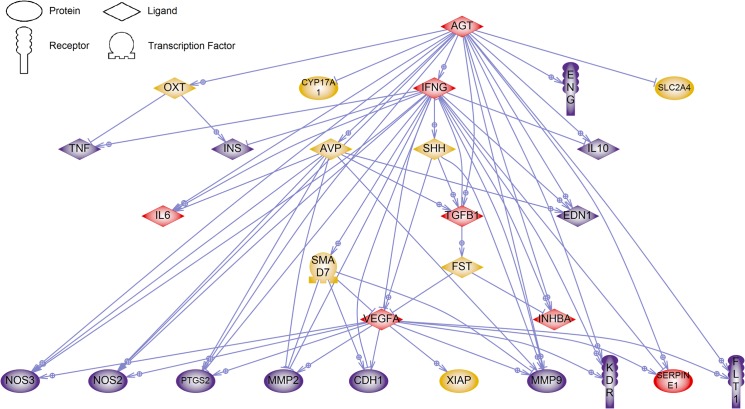
Downstream genes of identified regulators and targets of maternal PE susceptibility genes. A gene network of downstream targets of identified regulators/targets of maternal PE susceptibility genes was generated with Pathway Studio 9.0. Each link is supported by at least one published reference. Connecting genes are coloured in yellow, regulator/target genes of maternal PE susceptibility genes in red and major downstream targets of these genes in purple.

Pathways and interactions between susceptibility genes from the various functional groups were determined by an inbuilt literature-based database search in Pathway Studio 9.0. The major common pathway regulators and targets of susceptibility genes, with four or more connections, are *AGT*, *IFNG*, *IL6*, *INHBA*, *SERPINE1*, *TGFB1* and *VEGFA* (Fig 1). A similar analysis of the pathways and interactions between these major regulator and targets was then performed to identify their downstream genes that could serve as novel PE biomarkers. In total, 13 genes (*CDH1*, *EDN1*, *ENG*, *FLT1*, *IL10*, *INS*, *KDR*, *MMP2*, *MMP9*, *NOS2*, *NOS3*, *PTGS2* and *TNF*) downstream of these major regulators and targets were identified (Fig 2). Enrichment of the pathways associated with the susceptibility genes identified a total of 114 GO sets in 15 pathway categories (Table 2). The top three pathway categories were in the areas of reproduction, cell signalling and liver function. There were 10 pathway categories that were associated with at least two functional groups of susceptibility genes. All three functional groups of susceptibility genes were present in the pathway categories of neural function, differentiation and angiogenesis. Further details of these GO sets are presented as supplementary information in S1 Table.

**Table 2:** Categories of pathways associated with PE susceptibility genes.

Pathway categories	Genes involved	Number of GO sets
Reproduction	*ACVR1*, *ACVR1C*, *ACVR2A*, *INHA*, *INHBB*, *LNPEP*	31
Cell signalling	*ACVR1*, *ACVR1C*, *ACVR2A*, *INHA*, *INHBB*, *ERAP1*, *LNPEP*	21
Liver function	*ACVR1*, *ACVR1C*, *INHA*, *INHBB*, *LNPEP*	11
Immunity/Inflammation	*ACVR1*, *INHA*, *INHBB*, *ERAP1*, *ERAP2*	9
Neural function	*ACVR1C*, *INHA*, *COL4A1*, *COL4A2*, *LNPEP*	7
Protein modification	*ACVR1*, *ACVR1C*, *ACVR2A*, *INHA*, *LNPEP*	6
Apoptosis	*ACVR1*, *ACVR1C*, *INHA*, *INHBB*	5
Differentiation	*ACVR1*, *ACVR1C*, *INHA*, *INHBB*, *COL4A1*, *ERAP1*	5
Proliferation	*ACVR1*, *INHA*	5
Angiogenesis	*ACVR1*, *COL4A1*, *COL4A2*, *ERAP1*	4
Bone function	*ACVR1*, *ACVR2A*	3
Tissue remodelling	*COL4A2*, *ERAP1*, *ERAP2*, *LNPEP*	3
Blood pressure regulation	*ACVR2A*, *ERAP1*, *ERAP2*	2
Cardiovascular function	*ACVR1*	1
Transcription	*ACVR1*	1

### Major altered PE pathways

GSEA of the PE decidual transcriptome yielded 42 GO sets that were consistently altered between the two transcriptome profiling batches. The 13 pathway categories of these 42 differentially expressed GO sets (p<0.001) are presented in Table 3. The top three altered pathway categories were in the areas of immunity/inflammation, cell signalling and apoptosis, which represent 28 GO sets. Detailed information of the GO sets is available in S2 Table.

**Table 3:** Significantly altered pathway categories in PE decidual transcriptome (p < 0.001).

Pathway categories	Number of GO sets	p-value (Pathway Studio 9.0) ^a^	
		HT12	WG6
Immunity/inflammation	17	1.17 X 10^−20^–8.03 X 10^−4^	2.77 X 10^−12^–9.76 X 10^−4^
Cell signalling	7	3.71 X 10^−11^–4.22 X 10^−4^	4.55 X 10^−10^–6.30 X 10^−4^
Apoptosis	4	7.91 X 10^−10^–4.55 X 10^−4^	1.47 X 10^−8^–1.90 X 10^−5^
Adhesion	2	1.52 X 10^−6^–8.44 X 10^−4^	9.84 X 10^−8^–6.12 X 10^−6^
Cytoskeleton	2	3.53 X 10^−10^–5.48 X 10^−5^	7.50 X 10^−6^–2.59 X 10^−5^
Platelet function	2	8.00 X 10^−10^–6.10 X 10^−6^	6.06 X 10^−13^–2.91 X 10^−8^
Proliferation	2	6.10 X 10^−6^–8.56 X 10^−4^	6.07 X 10^−7^–2.53 X 10^−6^
Angiogenesis	1	1. 70 X 10^−8^	1.97 X 10^−8^
Liver function	1	9.44 X 10^−6^	3.62 X 10^−8^
Migration	1	5.96 X 10^−6^	1.77 X 10^−6^
Oxidative stress	1	1.06 X 10^−6^	1.11 X 10^−7^
Protein modification	1	5.09 X 10^−4^	5.13 X 10^−4^
Tissue remodelling	1	8.25 X 10^−4^	3.41 X 10^−5^

^a^ Presented as range where appropriate to reflect the spread of individual p-values of each Gene Ontology (GO) set from each transcriptome profiling batch.

### Overlap of PE transcriptome and PE susceptibility genes

The pathway categories of the GO sets that are concordant between the PE decidual transcriptome and the susceptibility genes are presented in Table 4. There were a total of 8 common GO sets that were grouped into five pathway categories. The top two pathway categories were apoptosis and cell signalling, which represent five GO sets. Details of the individual GO sets are available in S3 Table.

**Table 4:** Categories of pathways associated with PE susceptibility genes that are significantly altered in PE decidua (p < 0.001).

Pathway categories	Number of GO sets	p-value (Pathway Studio 9.0) ^a^	
		HT12	WG6
Apoptosis	3	7.91 X 10^−10^–4.55 X 10^−4^	1. 47 X 10^−8–^1.35 X 10^−5^
Cell signalling	2	7.35 X 10^−8^–1.73 X 10^−7^	4.55 X 10^−10^–2.23 X 10^−5^
Angiogenesis	1	1. 70 X 10^−8^	1.97 X 10^−8^
Liver function	1	9.44 X 10^−6^	3. 62 X 10^−8^
Tissue remodelling	1	8.25 X 10^−4^	3.41 X 10^−5^

^a^ Presented as range where appropriate to reflect the spread of individual p-values of each Gene Ontology (GO) set from each transcriptome profiling batch.

### Differentially expressed genes

From the ranking of the genes using the limma R package, none of the genes reached statistical significance when the stringent Bonferroni’s Correction (p < 1.26 X 10^6^) for multiple testing was applied. However, by using the semi-conservative statistical cut-off of p<0.001, a total of 8 differentially expressed genes were identified as concordant between the two datasets (Table 5). Downregulated genes were *CD72*, *DBP*, *DPP7*, *HS3ST2*, *PER3*, *SLC2A6* and *TNFRSF14*. PDK4 was the only upregulated gene. The entire list of genes with concordant differential expression between the two datasets is available in S4 Table.

**Table 5:** List of differentially expressed genes in PE decidua (p < 0.001).

Symbol	Illumina Probe_ID	Definition	Weighted fold change ^a^	p-value ^b^	
				HT12	WG6
HS3ST2	ILMN_1712475	Heparan sulfate (glucosamine) 3-O-sulfotransferase 2	-1.39	2.08 X 10^−5^	1. 83 X 10^−4^
TNFRSF14	ILMN_1697409	Tumor necrosis factor receptor superfamily, member 14	-1.35	1.74 X 10^−4^	8.93 X 10^−4^
SLC2A6	ILMN_1778321	Solute carrier family 2 (facilitated glucose transporter), member 6	-1.32	4.03 X 10^−4^	4.64 X 10^−4^
DPP7	ILMN_2252309	Dipeptidyl-peptidase 7	-1.22	1.36 X 10^−4^	2.32 X 10^−4^
CD72	ILMN_1723004	CD72 molecule	-1.21	7.09 X 10^−4^	1.41 X 10^−4^
PER3	ILMN_1660986	Period circadian clock 3	-1.20	4.85 X 10^−4^	1.37 X 10^−7^
DBP	ILMN_1715555	D site of albumin promoter (albumin D-box) binding protein	-1.20	3.82 X 10^−5^	9.65 X 10^−6^
PDK4	ILMN_1684982	Pyruvate dehydrogenase kinase, isozyme 4	1.72	3.29 X 10^−6^	1.23 X 10^−4^

^a^ Weighted to account for effect of the different sample sizes between each transcriptome profiling batch, values above below 0 indicate underexpression in PE relative to control, while values above 0 indicate overexpression in PE relative to control.

^b^ Presented as individual p-values from each transcriptome profiling batch.

## Discussion

In this study, we performed a novel genome-wide transcriptome directed pathway analysis of maternal PE susceptibility genes in a large set of 125 decidual samples. The transcriptome profiling yielded a total of only 8 differentially expressed genes. Two genes have reported associations with PE. *DBP*, an albumin promoter binding protein, was previously reported by Løset et al. [24] to be decreased in PE decidua and our results support this. In an earlier study of the fetal placenta, the other gene *SLC2A6*, which codes for a glucose transporter, was significantly increased in PE [39] and contrasts with our results where we found a decrease in PE maternal decidua. This may be reflective of the tissue being sampled from different parts of the maternal-fetal interface. The remaining genes have no known association with PE. However, this process of identifying differentially expressed genes that satisfy a statistically significant threshold may overlook other genes of equal or greater biological relevance; for example, smaller fold changes in the gene expression levels of several genes (an additive effect) in a common pathway may have a greater downstream impact compared with a large fold change in expression levels from a single gene [26]. By taking into account the decidual transcriptome, instead of focussing specifically on individual differentially expressed genes, there is increased power of detecting altered pathways in PE. Hence, by using our novel approach, we identified common altered pathways shared between microarray data and susceptibility genes.

To determine the interactions between the susceptibility genes from the various functional groups, we first constructed gene network pathways to identify common regulators and targets. There are many complex interactions between the genes. Some of these identified genes are both a regulator and target of the susceptibility genes. For example, one target of ERAP1 is AGT and AGT in turn is a regulator of ACVR2A. ERAP1 and ACVR2A are from different functional groups; however, we demonstrate an interactive link between these two genes via AGT, a finding that is not apparent from the traditional study approaches. These major regulators and targets are also implicated in previous PE studies. *AGT* is a major component of the renin-angiotensin-system, which regulates blood pressure and body-fluid volume, and has been widely investigated as a PE candidate gene [18]. Many studies have shown the remaining genes, which encode various cytokines, growth factors and protease inhibitors, are measurable in the maternal circulation and are significantly altered in PE. Activin A and inhibin A dimers derived from *INHBA*, IFNγ, IL6, plasminogen activator inhibitor-1, SERPINE1 and TGFβ1are all significantly increased in the maternal circulation in PE-affected women [40–45]. In contrast, VEGFA, an angiogenic factor, is significantly reduced in the circulation of women with PE [46].

Further analysis of downstream genes of the common regulators and targets was undertaken to identify potential PE biomarkers. With no selection bias, this analysis identified the anti-angiogenic factors, soluble FLT1 (sFLT) and endoglin (ENG), which are currently widely explored as predictive PE biomarkers and were first identified through microarray studies [47–49]. The other downstream genes also have recognised roles in PE. MMP2, MMP9 and CDH have altered expression in PE, with functional roles in the invasion of trophoblast cells into the maternal decidua [50–54]. *KDR* codes for a VEGF receptor that is significantly decreased in PE and may contribute to the endothelial dysfunction observed in PE [55]. Endothelin, which is coded by *EDN1*, is a vasoconstrictor that is significantly increased in the circulation of PE women [56]. Endothelin (EDN1), endothelial nitric oxide synthase (NOS3), inducible nitric oxide synthase (NOS2) and prostaglandin-endoperoxide synthase 2 (PTGS2) are well known for their involvement in maintaining blood pressure [57]. An IL10 null rodent model of PE was developed, highlighting a possible role for IL10 in PE [58]. Thus, these may be possible targets through which these PE susceptibility genes act to influence the development of PE and more targeted functional analyses can be performed with this knowledge. Therefore, a better understanding of how these different proteins interact may enable the development of a rigorous panel of PE biomarkers.

Additionally, the pathways associated with the susceptibility genes were determined and categorised. The majority of these pathway categories were associated with at least two functional groups of genes from the activin/inhibin signalling, structural components and M1 family aminopeptidases functional groups. The pathway categories of neural function, differentiation and angiogenesis had all three functional groups involved. This provides evidence that genes from distinct functional groups interact with each other and are involved in multiple pathways. Most, if not all of these pathway categories are thought to be important for a healthy, uncomplicated pregnancy. Therefore defects in multiple genes affecting several important pathways may promote PE susceptibility, providing additional weight behind the complex, multi-factorial nature of this serious disorder of obstetric medicine.

The top three altered PE pathway categories in the decidual transcriptome were immunity/inflammation, cell signalling and apoptosis, representing 28 altered gene sets, which were consistently altered between both transcriptome profiling batches. These pathway categories are consistent with the published literature [24, 59]. The top pathway category of immunity/inflammation supports the growing evidence of a highly dysregulated immune and inflammatory response at the PE maternal-fetal interface [60]. PE is hypothesised to be partly due to immune maladaptation to paternal antigens carried by the fetus, which leads to an exacerbated inflammatory response [61]. The disruption of cell signalling cascades that modulate many processes during pregnancy such as trophoblast invasion and spiral arteriole remodelling is hypothesised to lead to the shallow trophoblast invasion and poor spiral arteriole remodelling observed in PE pregnancies [62, 63]. Abnormal apoptosis regulation is also commonly observed in PE with alterations in multiple pathways such as the p53 pathway [64]. The concordant pathway categories between the susceptibility genes and the PE decidual transcriptome represent the altered pathways associated with the susceptibility genes. The top two concordant pathway categories of apoptosis and cell signalling, were also among the top three categories altered in the PE transcriptome. Therefore, the susceptibility genes may contribute to the development of PE through these particular pathways and focussing our functional analyses of the susceptibility genes in these areas will be of importance.

This integrative bioinformatics approach allows us to identify novel interactions and unbiased functional roles of the susceptibility genes. For example, the effect of altered collagen expression on blood pressure regulation through vasoactive factor production could be examined. Based on the gene networks, *COL4A1* regulates *VEGFA*, which in turn regulates many vasoactive factor genes such as *NOS3*, *NOS2*, *PTGS2* and *FLT1*. This novel function is not apparent from the known structural role of collagen. Interestingly, recent studies show that cleavage products derived from the non-collagenous domain of both COL4A1 and COL4A2, have significant anti-angiogenic effects on endothelial cells including increased apoptosis and decreased proliferation, and are being explored as novel cancer therapeutics [65–67]. Hence, this may be a plausible pathway through which collagen affects blood pressure regulation. The pathway category of blood pressure regulation was nominally altered (p < 0.05) in the PE decidual transcriptome. Therefore, undertaking this pathway-directed approach allows us to rationalise various studies that appear disparate, as the results from this study show that the genes identified through the different approaches interact with each other.

Given the complex genetics of PE, it is likely that the genes from other previously identified susceptibility loci, not present in our gene networks, may be part of a further extension of the current networks of gene interactions. Of the genes represented in the gene networks of this study, two genes *SHH* and *NOS3* reside at the 7q36 locus [28, 30]. The other loci identified thus far are at chromosomes 2p13, 2p25, 2q22, 9p13 and 10q22 [13, 29, 31, 68]. Further pathway analysis of these previously identified loci is warranted to extend the current knowledge.

In summary, we found that maternal PE susceptibility genes from distinct functional groups share similar downstream pathways through common regulators and targets. Downstream pathways associated with the susceptibility genes are altered in PE. Common pathways are the link between genes identified through the multiple approaches. An integrative bioinformatics approach allows us to identify novel interactions and unbiased functional roles of the susceptibility genes. Therefore, with this knowledge more targeted functional analyses of PE susceptibility genes in these key altered pathways can be performed to examine their contributions to the pathogenesis and severity of PE.

## Supporting Information

S1 Table Individual GO sets associated with maternal PE susceptibility genes. (XLSX)

S2 Table Individual GO sets altered in the PE decidual transcriptome. (XLSX)

S3 Table Individual GO sets concordant between the maternal PE susceptibility genes and the PE decidual transcriptome. (XLSX)

S4 Table Complete list of differentially expressed genes. (XLSX)

## References

[1] Duley L. The global impact of pre-eclampsia and eclampsia. Seminars in perinatology. 2009;33(3):130–7. 10.1053/j.semperi.2009.02.010 . 19464502

[2] Steegers EA, von Dadelszen P, Duvekot JJ, Pijnenborg R. Pre-eclampsia. The Lancet. 2010;376(9741):631–44. Epub 2010/07/06. S0140-6736(10)60279-6 [pii] 10.1016/S0140-6736(10)60279-6 . 20598363

[3] Meis PJ, Goldenberg RL, Mercer BM, Iams JD, Moawad AH, Miodovnik M, et al The preterm prediction study: risk factors for indicated preterm births. Maternal-Fetal Medicine Units Network of the National Institute of Child Health and Human Development. Am J Obstet Gynecol. 1998;178(3):562–7. Epub 1998/04/16. S0002937898704399 [pii] . 9539527 10.1016/s0002-9378(98)70439-9

[4] Saigal S, Doyle LW. An overview of mortality and sequelae of preterm birth from infancy to adulthood. Lancet. 2008;371(9608):261–9. Epub 2008/01/22. doi: 10.1016/S0140-6736(08)60136-1 S0140-6736(08)60136-1 [pii] . 18207020 10.1016/S0140-6736(08)60136-1

[5] Petrou S, Eddama O, Mangham L. A structured review of the recent literature on the economic consequences of preterm birth. Arch Dis Child Fetal Neonatal Ed. 2011;96(3):F225–32. Epub 2010/05/22. doi: 10.1136/adc.2009.161117 adc.2009.161117 [pii] . 20488863 10.1136/adc.2009.161117

[6] Anderson CM. Preeclampsia: exposing future cardiovascular risk in mothers and their children. Journal of Obstetric, Gynecologic, & Neonatal Nursing. 2007;36(1):3–8. Epub 2007/01/24. JOGN115 [pii] 10.1111/j.1552-6909.2006.00115.x . 17238941

[7] Brown DW, Dueker N, Jamieson DJ, Cole JW, Wozniak MA, Stern BJ, et al Preeclampsia and the risk of ischemic stroke among young women: results from the Stroke Prevention in Young Women Study. Stroke. 2006;37(4):1055–9. Epub 2006/02/18. 01.STR.0000206284.96739.ee [pii] 10.1161/01.STR.0000206284.96739.ee . 16484606

[8] Mangos GJ. Cardiovascular disease following pre-eclampsia: understanding the mechanisms. J Hypertens. 2006;24(4):639–41. Epub 2006/03/15. doi: 10.1097/01.hjh.0000217844.57466.85 00004872-200604000-00007 [pii] . 16531790 10.1097/01.hjh.0000217844.57466.85

[9] Nilsson E, Salonen Ros H, Cnattingius S, Lichtenstein P. The importance of genetic and environmental effects for pre-eclampsia and gestational hypertension: a family study. BJOG. 2004;111(3):200–6. Epub 2004/02/14. 14961879 10.1111/j.1471-0528.2004.00042x.x

[10] Salonen Ros H, Lichtenstein P, Lipworth L, Cnattingius S. Genetic effects on the liability of developing pre-eclampsia and gestational hypertension. Am J Med Genet. 2000;91(4):256–60. Epub 2000/04/15. 10.1002/(SICI)1096-8628(20000410)91:4<256::AID-AJMG3>3.0.CO;2-T [pii] . 10766979

[11] Johnson MP, Fitzpatrick E, Dyer TD, Jowett JB, Brennecke SP, Blangero J, et al Identification of two novel quantitative trait loci for pre-eclampsia susceptibility on chromosomes 5q and 13q using a variance components-based linkage approach. Molecular human reproduction. 2007;13(1):61–7. Epub 2006/11/07. 10.1093/molehr/gal095 . 17085769

[12] Moses EK, Fitzpatrick E, Freed KA, Dyer TD, Forrest S, Elliott K, et al Objective prioritization of positional candidate genes at a quantitative trait locus for pre-eclampsia on 2q22. Molecular human reproduction. 2006;12(8):505–12. Epub 2006/07/01. 10.1093/molehr/gal056 . 16809377

[13] van Dijk M, Mulders J, Poutsma A, Konst AA, Lachmeijer AM, Dekker GA, et al Maternal segregation of the Dutch preeclampsia locus at 10q22 with a new member of the winged helix gene family. Nat Genet. 2005;37(5):514–9. Epub 2005/04/05. ng1541 [pii] 10.1038/ng1541 . 15806103

[14] Chappell S, Morgan L. Searching for genetic clues to the causes of pre-eclampsia. Clin Sci (Lond). 2006;110(4):443–58. Epub 2006/03/11. CS20050323 [pii] 10.1042/CS20050323 . 16526948

[15] Zhao L, Bracken MB, DeWan AT. Genome-wide association study of pre-eclampsia detects novel maternal single nucleotide polymorphisms and copy-number variants in subsets of the Hyperglycemia and Adverse Pregnancy Outcome (HAPO) study cohort. Ann Hum Genet. 2013;77(4):277–87. Epub 2013/04/05. 10.1111/ahg.12021 23551011 PMC3740040

[16] Johnson MP, Brennecke SP, East CE, Goring HH, Kent JW Jr, Dyer TD, et al Genome-wide association scan identifies a risk locus for preeclampsia on 2q14, near the inhibin, beta B gene. PloS one. 2012;7(3):e33666 Epub 2012/03/21. 10.1371/journal.pone.0033666 22432041 PMC3303857

[17] Zhao L, Triche EW, Walsh KM, Bracken MB, Saftlas AF, Hoh J, et al Genome-wide association study identifies a maternal copy-number deletion in PSG11 enriched among preeclampsia patients. BMC Pregnancy Childbirth. 2012;12:61 Epub 2012/07/04. 1471-2393-12-61 [pii] 10.1186/1471-2393-12-61 22748001 PMC3476390

[18] Mutze S, Rudnik-Schoneborn S, Zerres K, Rath W. Genes and the preeclampsia syndrome. J Perinat Med. 2008;36(1):38–58. Epub 2008/01/11. 10.1515/JPM.2008.004 . 18184097

[19] Kleinrouweler CE, van Uitert M, Moerland PD, Ris-Stalpers C, van der Post JA, Afink GB. Differentially expressed genes in the pre-eclamptic placenta: a systematic review and meta-analysis. PloS one. 2013;8(7):e68991 Epub 2013/07/23. doi: 10.1371/journal.pone.0068991 PONE-D-13-12477 [pii] 23874842 10.1371/journal.pone.0068991 PMC3709893

[20] Louwen F, Muschol-Steinmetz C, Reinhard J, Reitter A, Yuan J. A lesson for cancer research: placental microarray gene analysis in preeclampsia. Oncotarget. 2012;3(8):759–73. Epub 2012/08/30. doi: 595 [pii] 22929622 10.18632/oncotarget.595 PMC3478454

[21] Eide IP, Isaksen CV, Salvesen KÅ, Langaas M, SchØNberg SA, Austgulen R. Decidual expression and maternal serum levels of heme oxygenase 1 are increased in pre-eclampsia. Acta Obstetricia et Gynecologica Scandinavica. 2008;87(3):272–9. 10.1080/00016340701763015 18307065

[22] Herse F, Dechend R, Harsem NK, Wallukat G, Janke J, Qadri F, et al Dysregulation of the Circulating and Tissue-Based Renin-Angiotensin System in Preeclampsia. Hypertension. 2007;49(3):604–11. 10.1161/01.hyp.0000257797.49289.71 17261642

[23] Lian IA, Loset M, Mundal SB, Fenstad MH, Johnson MP, Eide IP, et al Increased endoplasmic reticulum stress in decidual tissue from pregnancies complicated by fetal growth restriction with and without pre-eclampsia. Placenta. 2011;32(11):823–9. Epub 2011/09/13. doi: 10.1016/j.placenta.2011.08.005 S0143-4004(11)00417-6 [pii] 21907405 10.1016/j.placenta.2011.08.005 PMC3210381

[24] Loset M, Mundal SB, Johnson MP, Fenstad MH, Freed KA, Lian IA, et al A transcriptional profile of the decidua in preeclampsia. Am J Obstet Gynecol. 2011;204(1):84 e1-27. Epub 2010/10/12. doi: 10.1016/j.ajog.2010.08.043 S0002-9378(10)01098-7 [pii] 20934677 10.1016/j.ajog.2010.08.043 PMC3011026

[25] Winn VD, Gormley M, Paquet AC, Kjaer-Sorensen K, Kramer A, Rumer KK, et al Severe preeclampsia-related changes in gene expression at the maternal-fetal interface include sialic acid-binding immunoglobulin-like lectin-6 and pappalysin-2. Endocrinology. 2009;150(1):452–62. Epub 2008/09/27. doi: 10.1210/en.2008-0990 en.2008-0990 [pii] 18818296 10.1210/en.2008-0990 PMC2630905

[26] Subramanian A, Tamayo P, Mootha VK, Mukherjee S, Ebert BL, Gillette MA, et al Gene set enrichment analysis: a knowledge-based approach for interpreting genome-wide expression profiles. Proc Natl Acad Sci U S A. 2005;102(43):15545–50. Epub 2005/10/04. doi: 0506580102 [pii] 10.1073/pnas.0506580102 16199517 PMC1239896

[27] Founds SA, Conley YP, Lyons-Weiler JF, Jeyabalan A, Hogge WA, Conrad KP. Altered global gene expression in first trimester placentas of women destined to develop preeclampsia. Placenta. 2009;30(1):15–24. Epub 2008/11/26. doi: 10.1016/j.placenta.2008.09.015 S0143-4004(08)00314-7 [pii] 19027158 10.1016/j.placenta.2008.09.015 PMC2667803

[28] Arngrimsson R, Hayward C, Nadaud S, Baldursdottir A, Walker JJ, Liston WA, et al Evidence for a familial pregnancy-induced hypertension locus in the eNOS-gene region. Am J Hum Genet. 1997;61(2):354–62. Epub 1997/08/01. doi: S0002-9297(07)64061-0 [pii] 10.1086/514843 9311740 PMC1715904

[29] Arngrimsson R, Siguroardottir S, Frigge ML, Bjarnadottir RI, Jonsson T, Stefansson H, et al A genome-wide scan reveals a maternal susceptibility locus for pre-eclampsia on chromosome 2p13. Hum Mol Genet. 1999;8(9):1799–805. Epub 1999/08/11. doi: ddc220 [pii] . 10441346 10.1093/hmg/8.9.1799

[30] Guo G, Lade JA, Wilton AN, Moses EK, Grehan M, Fu Y, et al Genetic susceptibility to pre-eclampsia and chromosome 7q36. Hum Genet. 1999;105(6):641–7. Epub 2000/01/27. 10647900 10.1007/s004399900172

[31] Laivuori H, Lahermo P, Ollikainen V, Widen E, Haiva-Mallinen L, Sundstrom H, et al Susceptibility loci for preeclampsia on chromosomes 2p25 and 9p13 in Finnish families. Am J Hum Genet. 2003;72(1):168–77. Epub 2002/12/11. doi: S0002-9297(07)60515-1 [pii] 10.1086/345311 12474145 PMC378622

[32] Founds SA. Bridging global gene expression candidates in first trimester placentas with susceptibility loci from linkage studies of preeclampsia. J Perinat Med. 2011;39(4):361–8. Epub 2011/06/23. 10.1515/JPM.2011.045 . 21692683

[33] Jebbink J, Wolters A, Fernando F, Afink G, van der Post J, Ris-Stalpers C. Molecular genetics of preeclampsia and HELLP syndrome—a review. Biochim Biophys Acta. 2012;1822(12):1960–9. Epub 2012/08/25. 10.1016/j.bbadis.2012.08.004 . 22917566

[34] Yong HE, Murthi P, Borg A, Kalionis B, Moses EK, Brennecke SP, et al Increased decidual mRNA expression levels of candidate maternal pre-eclampsia susceptibility genes are associated with clinical severity. Placenta. 2014;35(2):117–24. 10.1016/j.placenta.2013.11.008 . 24331737 PMC4107207

[35] Brown MA, Gallery EDM, Gatt SP, Leslie G, Robinson J. Management of Hypertension in Pregnancy—Executive Summary. Med J Australia. 1993;158(10):700–2 . 8487690

[36] Brown MA, Hague WM, Higgins J, Lowe S, McCowan L, Oats J, et al The detection, investigation and management of hypertension in pregnancy: executive summary. Australian and New Zealand Journal of Obstetrics and Gynaecology. 2000;40(2):133–8. 10.1111/j.1479-828X.2000.tb01136.x 10925899

[37] Du P, Kibbe WA, Lin SM. lumi: a pipeline for processing Illumina microarray. Bioinformatics. 2008;24(13):1547–8. Epub 2008/05/10. 10.1093/bioinformatics/btn224 . 18467348

[38] Smyth GK. Linear models and empirical bayes methods for assessing differential expression in microarray experiments. Stat Appl Genet Mol Biol. 2004;3:Article3 Epub 2006/05/02. 10.2202/1544-6115.1027 . 16646809

[39] Chang SD, Chao AS, Peng HH, Chang YL, Wang CN, Cheng PJ, et al Analyses of placental gene expression in pregnancy-related hypertensive disorders. Taiwan J Obstet Gynecol. 2011;50(3):283–91. Epub 2011/10/28. doi: 10.1016/j.tjog.2011.07.005 S1028-4559(11)00126-4 [pii] . 22030040 10.1016/j.tjog.2011.07.005

[40] Kumar A, Begum N, Prasad S, Agarwal S, Sharma S. IL-10, TNF-alpha & IFN-gamma: potential early biomarkers for preeclampsia. Cell Immunol. 2013;283(1–2):70–4. Epub 2013/07/25. doi: 10.1016/j.cellimm.2013.06.012 S0008-8749(13)00106-8 [pii] . 23880295 10.1016/j.cellimm.2013.06.012

[41] Mori T, Shinohara K, Wakatsuki A, Watanabe K, Fujimaki A. Adipocytokines and endothelial function in preeclamptic women. Hypertens Res. 2010;33(3):250–4. Epub 2010/01/16. doi: 10.1038/hr.2009.222 hr2009222 [pii] . 20075929 10.1038/hr.2009.222

[42] Muttukrishna S, Knight PG, Groome NP, Redman CW, Ledger WL. Activin A and inhibin A as possible endocrine markers for pre-eclampsia. Lancet. 1997;349(9061):1285–8 . 9142063 10.1016/s0140-6736(96)09264-1

[43] Ozkan ZS, Simsek M, Ilhan F, Deveci D, Godekmerdan A, Sapmaz E. Plasma IL-17, IL-35, interferon-gamma, SOCS3 and TGF-beta levels in pregnant women with preeclampsia, and their relation with severity of disease. J Matern Fetal Neonatal Med. 2013. Epub 2013/11/02. 10.3109/14767058.2013.861415 . 24175856

[44] Prins JR, Gomez-Lopez N, Robertson SA. Interleukin-6 in pregnancy and gestational disorders. J Reprod Immunol. 2012;95(1–2):1–14. Epub 2012/07/24. doi: 10.1016/j.jri.2012.05.004 S0165-0378(12)00585-2 [pii] . 22819759 10.1016/j.jri.2012.05.004

[45] Silver HM, Lambert-Messerlian GM, Star JA, Hogan J, Canick JA. Comparison of maternal serum total activin A and inhibin A in normal, preeclamptic, and nonproteinuric gestationally hypertensive pregnancies. Am J Obstet Gynecol. 1999;180(5):1131–7. Epub 1999/05/18. doi: S0002937899002689 [pii] . 10329867 10.1016/s0002-9378(99)70606-x

[46] Sahay AS, Patil VV, Sundrani DP, Joshi AA, Wagh GN, Gupte SA, et al. A longitudinal study of circulating angiogenic and antiangiogenic factors and AT1-AA levels in preeclampsia. Hypertens Res. 2014. Epub 2014/04/11. doi: 10.1038/hr.2014.71 hr201471 [pii] . 24718301 10.1038/hr.2014.71

[47] Myatt L, Clifton RG, Roberts JM, Spong CY, Wapner RJ, Thorp JM Jr, et al Can changes in angiogenic biomarkers between the first and second trimesters of pregnancy predict development of pre-eclampsia in a low-risk nulliparous patient population? BJOG. 2013;120(10):1183–91. Epub 2013/01/22. 10.1111/1471-0528.12128 . 23331974 PMC4104359

[48] Maynard SE, Min JY, Merchan J, Lim KH, Li J, Mondal S, et al Excess placental soluble fms-like tyrosine kinase 1 (sFlt1) may contribute to endothelial dysfunction, hypertension, and proteinuria in preeclampsia. J Clin Invest. 2003;111(5):649–58. Epub 2003/03/06. 10.1172/JCI17189 12618519 PMC151901

[49] Venkatesha S, Toporsian M, Lam C, Hanai J, Mammoto T, Kim YM, et al Soluble endoglin contributes to the pathogenesis of preeclampsia. Nat Med. 2006;12(6):642–9. Epub 2006/06/06. doi: nm1429 [pii] 10.1038/nm1429 . 16751767

[50] Lockwood CJ, Oner C, Uz YH, Kayisli UA, Huang SJ, Buchwalder LF, et al Matrix metalloproteinase 9 (MMP9) expression in preeclamptic decidua and MMP9 induction by tumor necrosis factor alpha and interleukin 1 beta in human first trimester decidual cells. Biol Reprod. 2008;78(6):1064–72. Epub 2008/02/16. doi: 10.1095/biolreprod.107.063743 biolreprod.107.063743 [pii] 18276934 10.1095/biolreprod.107.063743 PMC3045968

[51] Palei AC, Sandrim VC, Amaral LM, Machado JS, Cavalli RC, Duarte G, et al Association between matrix metalloproteinase (MMP)-2 polymorphisms and MMP-2 levels in hypertensive disorders of pregnancy. Exp Mol Pathol. 2012;92(2):217–21. Epub 2012/02/14. doi: 10.1016/j.yexmp.2012.01.008 S0014-4800(12)00015-9 [pii] . 22327101 10.1016/j.yexmp.2012.01.008

[52] Plaks V, Rinkenberger J, Dai J, Flannery M, Sund M, Kanasaki K, et al Matrix metalloproteinase-9 deficiency phenocopies features of preeclampsia and intrauterine growth restriction. Proc Natl Acad Sci U S A. 2013;110(27):11109–14. Epub 2013/06/19. doi: 10.1073/pnas.1309561110 1309561110 [pii] 23776237 10.1073/pnas.1309561110 PMC3704020

[53] Zhang Z, Zhang L, Jia L, Cui S, Shi Y, Chang A, et al AP-2alpha suppresses invasion in BeWo cells by repression of matrix metalloproteinase-2 and -9 and up-regulation of E-cadherin. Mol Cell Biochem. 2013;381(1–2):31–9. Epub 2013/05/11. 10.1007/s11010-013-1685-8 . 23660954

[54] Li W, Mata KM, Mazzuca MQ, Khalil RA. Altered matrix metalloproteinase-2 and -9 expression/activity links placental ischemia and anti-angiogenic sFlt-1 to uteroplacental and vascular remodeling and collagen deposition in hypertensive pregnancy. Biochem Pharmacol. 2014;89(3):370–85. Epub 2014/04/08. doi: 10.1016/j.bcp.2014.03.017 S0006-2952(14)00204-4 [pii] . 24704473 10.1016/j.bcp.2014.03.017 PMC4034157

[55] Munaut C, Lorquet S, Pequeux C, Coulon C, Le Goarant J, Chantraine F, et al Differential expression of Vegfr-2 and its soluble form in preeclampsia. PloS one. 2012;7(3):e33475 Epub 2012/03/20. doi: 10.1371/journal.pone.0033475 PONE-D-11-12576 [pii] 22428059 10.1371/journal.pone.0033475 PMC3299790

[56] Aggarwal PK, Chandel N, Jain V, Jha V. The relationship between circulating endothelin-1, soluble fms-like tyrosine kinase-1 and soluble endoglin in preeclampsia. J Hum Hypertens. 2012;26(4):236–41. Epub 2011/04/01. doi: 10.1038/jhh.2011.29 jhh201129 [pii] . 21451568 10.1038/jhh.2011.29

[57] Bauer V, Sotnikova R. Nitric oxide—the endothelium-derived relaxing factor and its role in endothelial functions. Gen Physiol Biophys. 2010;29(4):319–40. Epub 2010/12/16. 21156995

[58] Kalkunte S, Boij R, Norris W, Friedman J, Lai Z, Kurtis J, et al Sera from preeclampsia patients elicit symptoms of human disease in mice and provide a basis for an in vitro predictive assay. Am J Pathol. 2010;177(5):2387–98. Epub 2010/10/05. doi: 10.2353/ajpath.2010.100475 S0002-9440(10)60291-X [pii] 20889559 10.2353/ajpath.2010.100475 PMC2966797

[59] Lian IA, Langaas M, Moses E, Johansson A. Differential gene expression at the maternal-fetal interface in preeclampsia is influenced by gestational age. PloS one. 2013;8(7):e69848 Epub 2013/08/13. 10.1371/journal.pone.0069848 23936112 PMC3729459

[60] Hsu P, Nanan RK. Innate and Adaptive Immune Interactions at the Fetal-Maternal Interface in Healthy Human Pregnancy and Pre-Eclampsia. Front Immunol. 2014;5:125 Epub 2014/04/16. 10.3389/fimmu.2014.00125 24734032 PMC3975095

[61] Erlebacher A. Immunology of the maternal-fetal interface. Annu Rev Immunol. 2013;31:387–411. Epub 2013/01/10. 10.1146/annurev-immunol-032712-100003 . 23298207

[62] Knofler M. Critical growth factors and signalling pathways controlling human trophoblast invasion. Int J Dev Biol. 2010;54(2–3):269–80. Epub 2009/10/31. doi: 10.1387/ijdb.082769mk 082769mk [pii] 19876833 10.1387/ijdb.082769mk PMC2974212

[63] Whitley GS, Cartwright JE. Trophoblast-mediated spiral artery remodelling: a role for apoptosis. J Anat. 2009;215(1):21–6. Epub 2009/02/14. doi: 10.1111/j.1469-7580.2008.01039.x JOA1039 [pii] 19215319 10.1111/j.1469-7580.2008.01039.x PMC2714635

[64] Sharp AN, Heazell AE, Baczyk D, Dunk CE, Lacey HA, Jones CJ, et al Preeclampsia is associated with alterations in the p53-pathway in villous trophoblast. PloS one. 2014;9(1):e87621 Epub 2014/02/06. doi: 10.1371/journal.pone.0087621 PONE-D-13-34686 [pii] 24498154 10.1371/journal.pone.0087621 PMC3907567

[65] Monboisse JC, Oudart JB, Ramont L, Brassart-Pasco S, Maquart FX. Matrikines from basement membrane collagens: a new anti-cancer strategy. Biochim Biophys Acta. 2014;1840(8):2589–98. Epub 2014/01/11. doi: 10.1016/j.bbagen.2013.12.029 S0304-4165(14)00002-6 [pii] . 24406397 10.1016/j.bbagen.2013.12.029

[66] Colorado PC, Torre A, Kamphaus G, Maeshima Y, Hopfer H, Takahashi K, et al Anti-angiogenic cues from vascular basement membrane collagen. Cancer Res. 2000;60(9):2520–6. Epub 2000/05/16. 10811134

[67] Kamphaus GD, Colorado PC, Panka DJ, Hopfer H, Ramchandran R, Torre A, et al Canstatin, a novel matrix-derived inhibitor of angiogenesis and tumor growth. J Biol Chem. 2000;275(2):1209–15. Epub 2000/01/08. 10625665 10.1074/jbc.275.2.1209

[68] Johnson MP, Brennecke SP, East CE, Dyer TD, Roten LT, Proffitt JM, et al Genetic dissection of the pre-eclampsia susceptibility locus on chromosome 2q22 reveals shared novel risk factors for cardiovascular disease. Molecular human reproduction. 2013;19(7):423–37. Epub 2013/02/20. doi: 10.1093/molehr/gat011 gat011 [pii] 23420841 10.1093/molehr/gat011 PMC3690803

